# Commensurate and Non-Commensurate Fractional-Order Discrete Models of an Electric Individual-Wheel Drive on an Autonomous Platform

**DOI:** 10.3390/e22030300

**Published:** 2020-03-05

**Authors:** Marcin Bąkała, Piotr Duch, J. A. Tenreiro Machado, Piotr Ostalczyk, Dominik Sankowski

**Affiliations:** 1 Institute of Applied Computer Science, ódź University of Technology, ul. Stefanowskiego 18/22, 90-537 Lodz, Poland; marcin.bakala@p.lodz.pl (M.B.); pduch@iis.p.lodz.pl (P.D.); piotr.ostalczyk@p.lodz.pl (P.O.); dsan@iis.p.lodz.pl (D.S.); 2 Institute of Engineering, Polytechnic of Porto, Rua Dr. António Bernardino de Almeida, 431, 4249-015 Porto, Portugal

**Keywords:** fractional-order backward-difference, difference equation, identification

## Abstract

This paper presents integer and linear time-invariant fractional order (FO) models of a closed-loop electric individual-wheel drive implemented on an autonomous platform. Two discrete-time FO models are tested: non-commensurate and commensurate. A classical model described by the second-order linear difference equation is used as the reference. According to the sum of the squared error criterion (SSE), we compare a two-parameter integer order model with four-parameter non-commensurate and three-parameter commensurate FO descriptions. The computer simulation results are compared with the measured velocity of a real autonomous platform powered by a closed-loop electric individual-wheel drive.

## 1. Introduction

Analysis of real dynamical systems based on the mathematical tools of fractional calculus [1,2,3,4,5,6,7] enables the construction of superior mathematical models [8,9,10,11,12], in the sense of providing a better match between the mathematical description and the measured data. Fractional modeling and control of an industrial selective compliant assembly robot arm was described in [13]. A fractional order PID controller was used for two-link robot control in [14]. A fractional order model of an inverted pendulum system based on simulated and experimental data was presented in [15]. Indeed, allowing any real orders in the differential or difference equations provides a good data fit, due to the assumed optimization criterion. On the other hand, there is an increase in the number of parameters which must be estimated. In this paper, non-commensurate and commensurate fractional order (FO) models are compared in terms of their effectiveness. Commensurate models are characterized by a smaller number of parameters. This is due to the parameter set required in the non-commensurate system, $\nu_{p},\nu_{p-1},\ldots ,\nu_{1}\in R$, as opposed to that in the commensurate system, pν,(p−1)ν,…,ν, ν∈R, p∈N. In each case, the number of first order differential equation (FODE) coefficients is the same. This paper provides a short introduction to the non-commensurate and commensurate systems described by linear FO difference equations and their state-space forms [16]. We then describe the closed-loop DC individual-wheel drive implemented in an autonomous platform. We propose two simple linear models based on the FODE. The simulation results are compared with the measured data. Similar results were obtained using the two considered models. However, commensurate models have only one multiple order. The paper is organized as follows. In Section 2, fundamental information concerning the variable, fractional order backward difference equation is introduced. Section 3 describes the closed-loop individual-wheel DC drive motor. In Section 4, the results provided by the proposed models are compared with the measured velocity of an electrical set-up, according to sum of the squared error criterion (SSE). The results are discussed in Section 5.

## 2. Non-Commensurate and Commensurate Difference Equation

Mathematical models of dynamical systems can be expressed by means of the following differential equations:

(1) $$\begin{array}{c} F\left[\frac{d^{p}y\left(t\right)}{dt^{p}},\frac{d^{p-1}y\left(t\right)}{dt^{p-1}},\ldots ,\frac{y(t)}{dt},y\left(t\right),\right.\\ \left.\frac{d^{q}u\left(t\right)}{dt^{q}},\frac{d^{q-1}u\left(t\right)}{dt^{q-1}},\ldots ,\frac{u(t)}{dt},u\left(t\right),t\right]=0, \end{array}$$

where y(t) and u(t) denote the output and the input signals, respectively, and function *F* is non-linear in general. One can also include FO derivatives in the models:

(2) $$\begin{array}{c} F\left[{}_{t_{0}}^{GL}D_{t}^{\nu_{p}}y\left(t\right){,}_{t_{0}}^{GL}D_{t}^{\nu_{p-1}}y\left(t\right),\ldots {,}_{t_{0}}^{GL}D_{t}^{\nu_{1}}y\left(t\right),y\left(t\right),\right.\\ \left.{}_{t_{0}}^{GL}D_{t}^{\mu_{q}}u\left(t\right),\ldots {,}_{t_{0}}^{GL}D_{t}^{\mu_{1}}u\left(t\right),u\left(t\right),t\right]=0, \end{array}$$

where ${}_{t_{0}}^{GL}D_{t}^{\nu_{p}}y\left(t\right)$ denotes the Gr$\ddot{u}$nwald-Letnikov FO derivative. Let all orders be arranged in a series such that $\nu_{p}>\nu_{p-1}>\ldots >\nu_{1}>0$ and $\mu_{q}>\mu_{q-1}>\ldots >\mu_{1}>0$.

Assume that all orders can be expressed in the form

(3) $$\nu_{i}=\frac{e_{i}}{d_{i}}\;\mathrm{for}\;i=1,2,\ldots ,p,\;\mathrm{and}\;e_{i},d_{i}\in Z_{+}$$

(4) $$\mu_{j}=\frac{g_{j}}{f_{j}}\;\mathrm{for}\;j=1,2,\ldots ,q,\;\mathrm{and}\;g_{j},f_{j}\in Z_{+}.$$

One can substitute the Gr$\ddot{u}$nwald-Letnikov fractional left derivative for the Gr$\ddot{u}$nwald-Letnikov fractional order backward difference (GL-FOBD). Let us define the finite sum for $\nu \in R_{+}$

(5) $$\begin{array}{c} {}_{k_{0}}^{GL}\Delta_{k}^{\left(\nu \right)}f\left(k\right)=\sum_{i=k_{0}}^{k}a^{\left(\nu \right)}\left(i-k_{0}\right)f\left(k+k_{0}-i\right)\\ =\sum_{i=0}^{k-k_{0}}a^{\left(\nu \right)}\left(i\right)f\left(k-i\right), \end{array}$$

where

(6) $$a^{\left(\nu \right)}\left(k\right)=\left\{\begin{array}{ccc} 0 & \mathrm{for} & k<0\\ 1 & \mathrm{for} & k=0\\ {\left(-1\right)}^{k}\frac{\nu (\nu -1)\ldots (\nu -k+1)}{k!} & \mathrm{for} & k=1,2,3,\ldots \end{array}\right.$$

The GL-FOBD divided by the sampling time *h* (which should be relatively small in real-world applications) in Equation (2) approximates the derivatives, yielding:

(7) $$\begin{array}{c} {}_{t_{0}}^{GL}D_{t}^{\left(\nu_{i}\right)}y\left(t\right)\approx \frac{{}_{k_{0}h}^{GL}\Delta_{kh}^{\left(\nu_{i}\right)}y\left(kh\right)}{h^{\nu_{i}}},\\ {}_{t_{0}}^{GL}D_{t}^{\left(\mu_{j}\right)}u\left(t\right)\approx \frac{{}_{k_{0}h}^{GL}\Delta_{kh}^{\left(\mu_{j}\right)}u\left(kh\right)}{h^{\mu_{j}}}. \end{array}$$

Hence, for i=1,2,…,p, j=1,2,…,q, from (2) one obtains the FODE

(8) $$\begin{array}{c} F\left[{}_{k_{0}h}^{GL}\Delta_{kh}^{\left(\nu_{p}\right)}y\left(kh\right),\ldots {,}_{k_{0}h}^{GL}\Delta_{kh}^{\left(\nu_{1}\right)}y\left(kh\right),y\left(kh\right),\right.\\ \left.{}_{k_{0}h}^{GL}\Delta_{kh}^{\left(\mu_{q}\right)}u\left(kh\right),\ldots {,}_{k_{0}h}^{GL}\Delta_{kh}^{\left(\mu_{1}\right)}u\left(kh\right),u\left(kh\right),kh\right]=0. \end{array}$$

Let *d* be the least common denominator of fractions (3) and (4). Then, the FO takes the form

(9) $$\nu_{i}=\frac{n_{i}}{d}\;\mathrm{for}\;i=1,2,\ldots ,p,\;\mathrm{and}\;\;n_{i},d\in Z_{+}$$

(10) $$\mu_{j}=\frac{m_{j}}{d}\;\mathrm{for}\;j=1,2,\ldots ,q,\;\mathrm{and}\;\;m_{j},d\in Z_{+}$$

where

(11) $$\nu =\frac{1}{d}.$$

If we introduce the notation

(12) $${}_{k_{0}h}^{GL}\Delta_{kh}^{\left(\frac{1}{d}\right)}y\left(kh\right){=}_{k_{0}h}^{GL}\Delta_{kh}^{\left(\nu \right)}y\left(kh\right),$$

(13) $${}_{k_{0}h}^{GL}\Delta_{kh}^{\left(\frac{1}{d}\right)}u\left(kh\right){=}_{k_{0}h}^{GL}\Delta_{kh}^{\left(\nu \right)}u\left(kh\right),$$

then the appropriate FOBD is as follows

(14) $$\begin{array}{c} {}_{k_{0}h}^{GL}\Delta_{kh}^{\left(\nu_{i}\right)}y\left(kh\right){=}_{k_{0}h}^{GL}\Delta_{kh}^{\left(n_{i}\nu \right)}y\left(kh\right)\\ =\left[\underset{n_{i}}{\underset{︸}{{}_{k_{0}h}^{GL}\Delta_{kh}^{\left(\nu \right)}{}_{k_{0}h}^{GL}\Delta_{kh}^{\left(\nu \right)}\ldots {}_{k_{0}h}^{GL}\Delta_{kh}^{\left(\nu \right)}}}\right]y\left(kh\right). \end{array}$$

Under the above transformations, the FODE (8) takes the form

(15) $$\begin{array}{c} F\left[{}_{k_{0}h}^{GL}\Delta_{kh}^{\left(\nu p\right)}y\left(kh\right),\ldots {,}_{k_{0}h}^{GL}\Delta_{kh}^{\left(\nu \right)}y\left(kh\right),y\left(kh\right),\right.\\ \left.{}_{k_{0}h}^{GL}\Delta_{kh}^{\left(\nu q\right)}u\left(kh\right),\ldots {,}_{k_{0}h}^{GL}\Delta_{kh}^{\left(\nu \right)}u\left(kh\right),u\left(kh\right),kh\right]=0. \end{array}$$

### 2.1. Non-Commensurate and Commensurate Linear Time-Invariant FODE

Linear time-invariant FOBDs constitute a special but very important class of FOBD. With a non-linear FODE, one can apply the linearization procedure around steady-state conditions, under the assumption of a relatively small change in the input signal. This is the first approach in system modeling. The linear time invariant non-commensurate FODE takes the form

(16) $$\sum_{i=0}^{p}{a_{i}}_{k_{0}}^{GL}\Delta_{k}^{\left(\nu_{i}\right)}y\left(k\right)=\sum_{j=0}^{q}{b_{j}}_{k_{0}}^{GL}\Delta_{k}^{\left(\nu_{j}\right)}u\left(k\right)=v\left(k\right),$$

where the FO are ordered in the same way as in (8). The linear time-invariant commensurate FODE is as follows:

(17) $$\sum_{i=0}^{p}{a_{i}}_{k_{0}}^{GL}\Delta_{k}^{\left(i\nu \right)}y\left(k\right)=\sum_{j=0}^{q}{b_{j}}_{k_{0}}^{GL}\Delta_{k}^{\left(j\nu \right)}u\left(k\right)=v\left(k\right),$$

where $a_{i},b_{j}$ are constant coefficients and $a_{p}=1$, p≥q, and u(k) is a known input signal. For d=1 in (11), i.e., for ν=1, the considered FODE represents the classical integer-order difference equations (IODEs).

Every rational order non-commensurate system can be approximated by a commensurate system. The transformation procedure is described in [16].

### 2.2. State-Space Equations of the Non-Commensurate and Commensurate Systems

The FOBD has a concatenation property

(18) $${}_{k_{0}}^{GL}\Delta_{k}^{\left(\nu \right)}\left[{}_{k_{0}}^{GL}\Delta_{k}^{\left(\mu \right)}y\left(k\right)\right]{=}_{k_{0}}^{GL}\Delta_{k}^{\left(\nu +\mu \right)}y\left(k\right),$$

for ν,μ≥0. Therefore, every FOBD in Equation (16) can be expressed in the form

(19) $${}_{k_{0}}^{GL}\Delta_{k}^{\left(\nu_{i}\right)}y\left(k\right)=\prod_{k=0}^{i}{}_{k_{0}}^{GL}\Delta_{k}^{\left(\nu_{i}-\nu_{i-1}\right)}y\left(k\right),$$

when $\nu_{0}=0$. Note that all orders verify the conditions $\nu_{i}-\nu_{i-1}>0$ for i=p,p−1,…,2,1. Hence, the FODE takes the form

(20) $$\sum_{i=0}^{p}a_{i}\prod_{k=0}^{i}{}_{k_{0}}^{GL}\Delta_{k}^{\left(\nu_{i}-\nu_{i-1}\right)}y\left(k\right)=v\left(k\right).$$

One may now define new variables, referred to henceforth as state-variables

(21) $$\begin{array}{c} y\left(k\right)=x_{1}\left(k\right)\\ {}_{k_{0}}^{GL}\Delta_{k}^{\left(\nu_{1}\right)}y\left(k\right){=}_{k_{0}}^{GL}\Delta_{k}^{\left(\nu_{1}\right)}x_{1}\left(k\right)=x_{2}\left(k\right)\\ {}_{k_{0}}^{GL}\Delta_{k}^{\left(\nu_{2}-\nu_{1}\right)}\left[{}_{k_{0}}^{GL}\Delta_{k}^{\left(\nu_{1}\right)}y\left(k\right)\right]{=}_{k_{0}}^{GL}\Delta_{k}^{\left(\nu_{2}-\nu_{1}\right)}x_{2}\left(k\right)=x_{3}\left(k\right)\\ \vdots \\ {}_{k_{0}}^{GL}\Delta_{k}^{\left(\nu_{p-1}-\nu_{p-2}\right)}x_{p-1}\left(k\right)=x_{p}\left(k\right) \end{array}$$

The introduction of the above set of state variables into (20) gives the equation

(22) $${}_{k_{0}}^{GL}\Delta_{k}^{\left(\nu_{p}-\nu_{p-1}\right)}x_{p}\left(k\right)+\sum_{i=1}^{p-1}a_{i}x_{i}\left(k\right)=v\left(k\right).$$

Equations (21) and (22) can be expressed in matrix-vector form

(23) $$\begin{array}{c} {}_{k_{0}}^{GL}\Delta_{k}^{\left(\nu \right)}x\left(k\right)=Ax\left(k\right)+bv\left(k\right), \end{array}$$

(24) $$\begin{array}{c} y(k)=cx(k)+bv(k), \end{array}$$

where

(25) $${}_{k_{0}}^{GL}\Delta_{k}^{\left(\nu \right)}x\left(k\right)=\left[\begin{array}{c} {}_{k_{0}}^{GL}\Delta_{k}^{\left(\nu_{1}\right)}x_{1}\left(k\right)\\ {}_{k_{0}}^{GL}\Delta_{k}^{\left(\nu_{2}-\nu_{1}\right)}x_{2}\left(k\right)\\ {}_{k_{0}}^{GL}\Delta_{k}^{\left(\nu_{3}-\nu_{2}\right)}x_{3}\left(k\right)\\ \vdots \\ {}_{k_{0}}^{GL}\Delta_{k}^{\left(\nu_{p-1}-\nu_{p-2}\right)}x_{p-1}\left(k\right)\\ {}_{k_{0}}^{GL}\Delta_{k}^{\left(\nu_{p}-\nu_{p-1}\right)}x_{p}\left(k\right) \end{array}\right],$$

(26) $$\nu =\left[\begin{array}{c} \nu_{1}\\ \nu_{2}-\nu_{1}\\ \nu_{3}-\nu_{2}\\ \vdots \\ \nu_{p-1}-\nu_{p-2}\\ \nu_{p}-\nu_{p-1} \end{array}\right],\;x\left(k\right)=\left[\begin{array}{c} x_{1}\left(k\right)\\ x_{2}\left(k\right)\\ x_{3}\left(k\right)\\ \vdots \\ x_{p-1}\left(k\right)\\ x_{p}\left(k\right) \end{array}\right]$$

(27) $$A=\left[\begin{array}{cccccc} 0 & 1 & 0 & \ldots & 0 & 0\\ 0 & 0 & 1 & \ldots & 0 & 0\\ 0 & 0 & 0 & \ldots & 0 & 0\\ \vdots \\ 0 & 0 & 0 & \ldots & 0 & 1\\ -a_{0} & -a_{1} & -a_{2} & \ldots & -a_{p-2} & -a_{p-1} \end{array}\right],\;b=\left[\begin{array}{c} 0\\ 0\\ 0\\ \vdots \\ 0\\ 1 \end{array}\right],$$

(28) $$c=\left[\begin{array}{cccccc} 0 & 0 & 0 & \ldots & 0 & 1, \end{array}\right]\;d=\left[1\right].$$

In the case of the commensurate system, we have $\nu_{i}=i\nu$ for i=1,2,…,p. Hence, $\nu_{i}-\nu_{i-1}=\nu$ and the left-hand side vector (25) simplify to

(29) $${}_{k_{0}}^{GL}\Delta_{k}^{\left(\nu \right)}x\left(k\right)=\left[\begin{array}{c} {}_{k_{0}}^{GL}\Delta_{k}^{\left(\nu \right)}x_{1}\left(k\right)\\ {}_{k_{0}}^{GL}\Delta_{k}^{\left(\nu \right)}x_{2}\left(k\right)\\ {}_{k_{0}}^{GL}\Delta_{k}^{\left(\nu \right)}x_{3}\left(k\right)\\ \vdots \\ {}_{k_{0}}^{GL}\Delta_{k}^{\left(\nu \right)}x_{p-1}\left(k\right)\\ {}_{k_{0}}^{GL}\Delta_{k}^{\left(\nu \right)}x_{p}\left(k\right) \end{array}\right]{=}_{k_{0}}^{GL}\Delta_{k}^{\left(\nu \right)}\left[\begin{array}{c} x_{1}\left(k\right)\\ x_{2}\left(k\right)\\ x_{3}\left(k\right)\\ \vdots \\ x_{p-1}\left(k\right)\\ x_{p}\left(k\right) \end{array}\right]$$

## 3. Closed-Loop DC Individual-Wheel Drive

**Figure 1:**
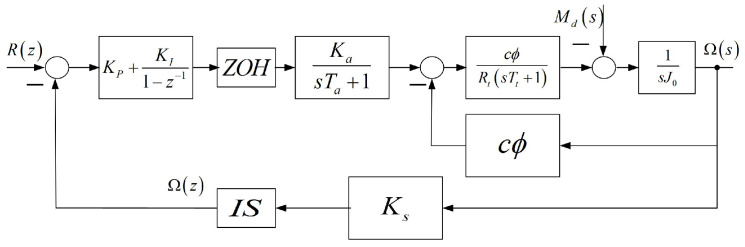
Block diagram of the closed-loop electric individual-wheel drive.

**Figure 2:**
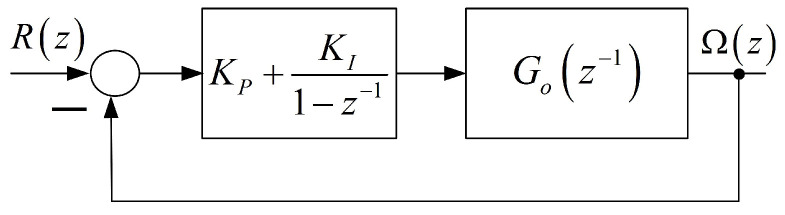
Block diagram of the discrete closed-loop electric individual-wheel drive.

The block diagram [17] of the closed-loop electrical drive [18,19] is shown in Figure 1, where R(z) and Ω(z) are the discrete-time reference and output (angular velocity) signals, and $K_{P}$ and $K_{I}$ denote the PI controller parameters. The acronyms ZOH and IS stand for zero-order hold and the ideal sampler, respectively. The PWM inverter is represented by a first-order inertial element with parameters $K_{a}$ and $T_{a}$. The DC motor is characterized by the electrical time constant $T_{t}$ and by the parameter cΦ, which denotes the back-EMF and torque constant of the motor. The symbols $J_{o}$ and $K_{s}$ stand for the motor plus load inertia and the sensor coefficient, respectively. The wheel is suspended in the air, which means that the external disturbance moment $M_{d}\left(s\right)=0$. Under the considered conditions of the experiment, the internal friction should also be taken into account [20]. The continuous part of the closed-loop system is described by the transfer function

(30) $$G_{o}\left(s\right)=\frac{\frac{K_{a}\left(1-e^{-sh}\right)}{c\Phi }}{s\left(T_{t}T_{m}s^{2}+T_{m}+1\right)\left(T_{a}s+1\right)},$$

where *h* is the sampling time and $T_{m}$ is the mechanical time constant. Its discrete counterpart may be expressed as

(31) $$G_{o}\left(z^{-1}\right)=\frac{K_{d}\left(T_{1}^{\prime }-z^{-1}\right)\left(T_{2}^{\prime }-z^{-1}\right)}{\left(T_{1}-z^{-1}\right)\left(T_{2}-z^{-1}\right)\left(T_{3}-z^{-1}\right)},$$

where $T_{i}$ and $T_{j}^{\prime }$ for i=1,2,3 and j=1,2, are time constants related to the closed-loop system parameters mentioned above. The discrete closed-loop system is shown in Figure 2. From this block diagram, one obtains the discrete transfer function in the form

(32) $$G_{c}\left(z^{-1}\right)=\frac{B_{0}+B_{1}z^{-1}+B_{2}z^{-2}+B_{3}z^{-3}}{1+A_{1}z^{-1}+A_{2}z^{-2}+A_{3}z^{-3}+A_{4}z^{-4}}.$$

Related to the above discrete transfer function, there is the commensurate IODE (17) with p=4,q=3 and coefficients $a_{i}$, $b_{0}$, $b_{i}$, i=0,…,3, which are functions of $A_{i},B_{j}$, i=1,…,4,j=0,…,3. Hence, the model resulting from the block diagram in Figure 2 contains eight parameters.

**Figure 3:**
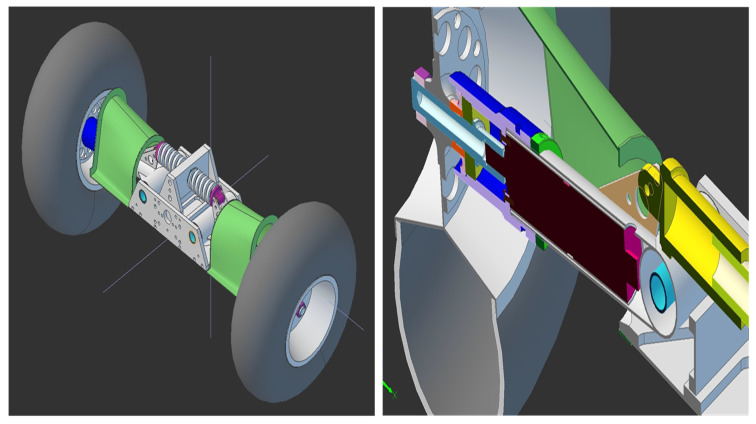
Model 3D of the autonomous platform wheel.

**Figure 4:**
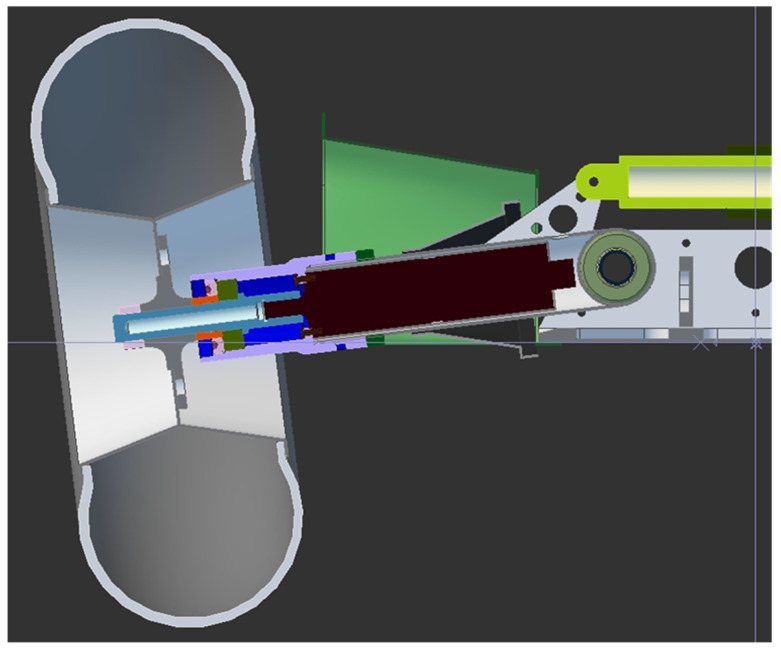
Model of the autonomous wheel platform—2D view.

**Figure 5:**
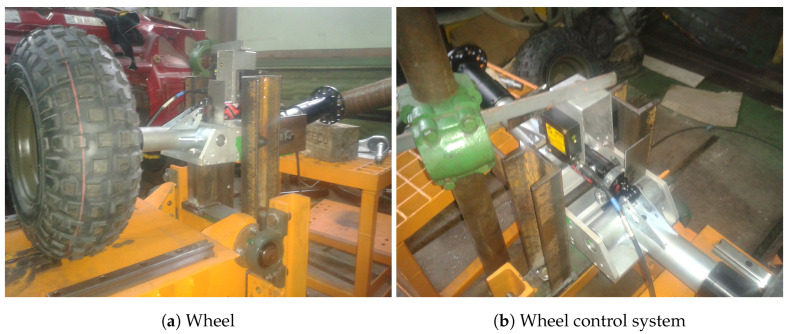
Photographs of the autonomous wheel platform.

Figure 3 and Figure 4 present 3D drawings of the considered dynamical system. Figure 5 shows the experimental set-up. Note that in the identification procedure the main external disturbance is assumed to be zero d(k)=0.

### 3.1. Transient Characteristics of Measured DC Motor Wheel Drive

**Figure 6:**
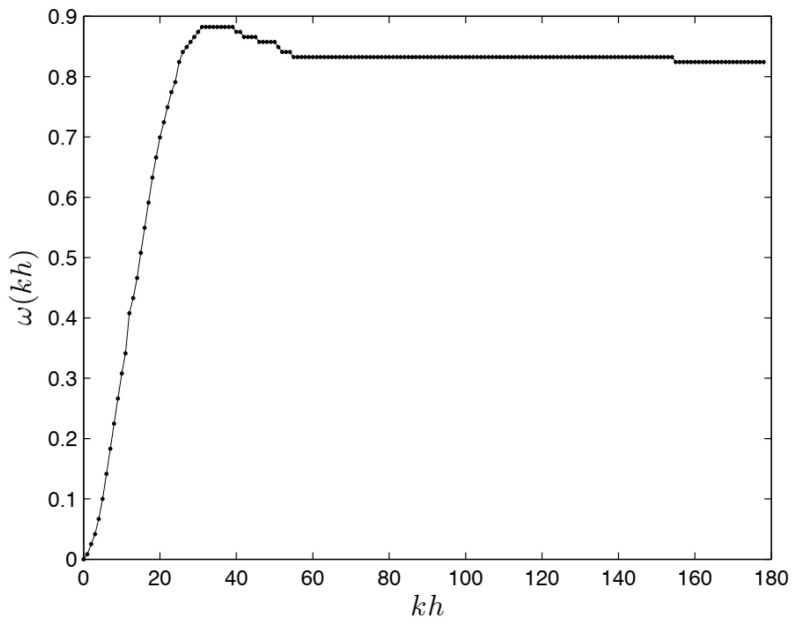
Measured angular velocity of wheel-drive system under step excitation.

The discrete step response presented in Figure 6 suggests the need for second-order-damped oscillation models. Evidently, in the “black box” measured data there are hidden non-linear frictions and external disturbance moments which the non-commensurate and commensurate FODE models should describe.

### 3.2. Classical Two-Parameter Linear Integer-Order Difference Equation Model of the Wheel-Drive

As mentioned previously, the classical oscillation model takes (17) with p=2,q=0, $\nu_{2}=2$ and $\nu_{1}=1$. This assumption means that the model is described by the IODE in the form

(33) $${}_{k_{0}}^{GL}\Delta_{k}^{\left(2\right)}y_{1,2}\left(k\right)+a_{1}{}_{k_{0}}^{GL}\Delta_{k}^{\left(1\right)}y_{1,2}\left(k\right)+a_{0}y_{1,2}\left(k\right)=a_{0}r\left(k\right).$$

Without lack of generality one can assume $b_{0}=a_{0}$. Hence, we obtain two parameters of the model, $a_{1}$ and $a_{0}$. The integer orders are two and one. The related state-space form is as follows:

(34) $$\begin{array}{c} {}_{k_{0}}^{GL}\Delta_{k}^{\left(1\right)}\left[\begin{array}{c} x_{1}\left(k\right)\\ x_{2}\left(k\right) \end{array}\right]=\left[\begin{array}{cc} 0 & 1\\ -a_{0} & -a_{1} \end{array}\right]+\left[\begin{array}{c} 0\\ 1 \end{array}\right]r\left(k\right),\\ y_{1,2}\left(k\right)=\left[\begin{array}{cc} 1 & 0 \end{array}\right]\left[\begin{array}{c} x_{1}\left(k\right)\\ x_{2}\left(k\right) \end{array}\right]+\left[a_{0}\right]r\left(k\right). \end{array}$$

### 3.3. Non-Commensurate Three-Parameter Linear Fractional-Order Difference Equation Model of the Wheel-Drive

As a special case of (16), one assumes a three-parameter model

(35) $$\begin{array}{c} {}_{k_{0}}^{GL}\Delta_{k}^{\left(\nu_{2}\right)}y_{\nu_{1},\nu_{2}}\left(k\right)+a_{1}{}_{k_{0}}^{GL}\Delta_{k}^{\left(\nu_{1}\right)}y_{\nu_{1},\nu_{2}}\left(k\right)\\ +a_{0}y_{\nu_{1},\nu_{2}}\left(k\right)=a_{0}r\left(k\right). \end{array}$$

Related state-space equations are

(36) $$\begin{array}{c} \left[\begin{array}{c} {}_{k_{0}}^{GL}\Delta_{k}^{\left(\nu_{1}\right)}x_{1}\left(k\right)\\ {}_{k_{0}}^{GL}\Delta_{k}^{\left(\nu_{2}-\nu_{1}\right)}x_{2}\left(k\right) \end{array}\right]=\left[\begin{array}{cc} 0 & 1\\ -a_{0} & -a_{1} \end{array}\right]+\left[\begin{array}{c} 0\\ 1 \end{array}\right]r\left(k\right),\\ y_{\nu_{1},\nu_{2}}\left(k\right)=\left[\begin{array}{cc} 1 & 0 \end{array}\right]\left[\begin{array}{c} x_{1}\left(k\right)\\ x_{2}\left(k\right) \end{array}\right]+\left[a_{0}\right]r\left(k\right) \end{array}$$

with four unknown parameters, $a_{1}$, $a_{0}$, $\nu_{2}$ and $\nu_{1}$.

### 3.4. Commensurate Linear Fractional-Order State-Space Model of the Wheel-Drive

The FODE has three parameters, $a_{1},$$a_{0}$ and $\nu \in R_{+}$.

(37) $$\begin{array}{c} {}_{k_{0}}^{GL}\Delta_{k}^{\left(2\nu \right)}y_{\nu ,2\nu }\left(k\right)+a_{1}{}_{k_{0}}^{GL}\Delta_{k}^{\left(\nu \right)}y_{\nu ,2\nu }\left(k\right)+a_{0}y_{\nu ,2\nu }\left(k\right)\\ =a_{0}r\left(k\right), \end{array}$$

(38) $$\begin{array}{c} {}_{k_{0}}^{GL}\Delta_{k}^{\left(\nu \right)}\left[\begin{array}{c} x_{1}\left(k\right)\\ x_{2}\left(k\right) \end{array}\right]=\left[\begin{array}{cc} 0 & 1\\ -a_{0} & -a_{1} \end{array}\right]+\left[\begin{array}{c} 0\\ 1 \end{array}\right]r\left(k\right),\\ y_{\nu ,2\nu }\left(k\right)=\left[\begin{array}{cc} 1 & 0 \end{array}\right]\left[\begin{array}{c} x_{1}\left(k\right)\\ x_{2}\left(k\right) \end{array}\right]+\left[a_{0}\right]r\left(k\right). \end{array}$$

## 4. Comparison of Models

The optimal choice of parameters related to the three linear structures is described by formulas (30), (32) and (34), which are based on the minimization of the performance index sum of the squared errors (SSE). Let us denote the measured output signal as $y_{m}\left(k\right)$. If one defines three error functions

(39) $$\begin{array}{c} e_{1,2}\left(k\right)=y_{m}\left(k\right)-y_{1,2}\left(k\right), \end{array}$$

(40) $$\begin{array}{c} e_{\nu_{1},2\nu_{1}}\left(k\right)=y_{m}\left(k\right)-y_{\nu_{1},2\nu_{1}}\left(k\right), \end{array}$$

(41) $$\begin{array}{c} e_{\nu_{1},\nu_{2}}\left(k\right)=y_{m}\left(k\right)-y_{\nu_{1},\nu_{2}}\left(k\right), \end{array}$$

then the criteria are the functions

(42) $$\begin{array}{c} SSE\left(a_{0},a_{1}\right)=\sum_{k=0}^{k_{max}}e_{1,2}^{2}\left(k\right), \end{array}$$

(43) $$\begin{array}{c} SSE\left(a_{0},a_{1},\nu_{1},\nu_{2}\right)=\sum_{k=0}^{k_{max}}e_{\nu_{1},\nu_{2}}^{2}\left(k\right), \end{array}$$

(44) $$\begin{array}{c} SSE\left(a_{0},a_{1},\nu_{1}\right)=\sum_{k=0}^{k_{max}}e_{\nu_{1},2\nu_{1}}^{2}\left(k\right). \end{array}$$

Numerical tests found minimum values for the coefficients and FO, which are listed in Table 1.

**Figure 7:**
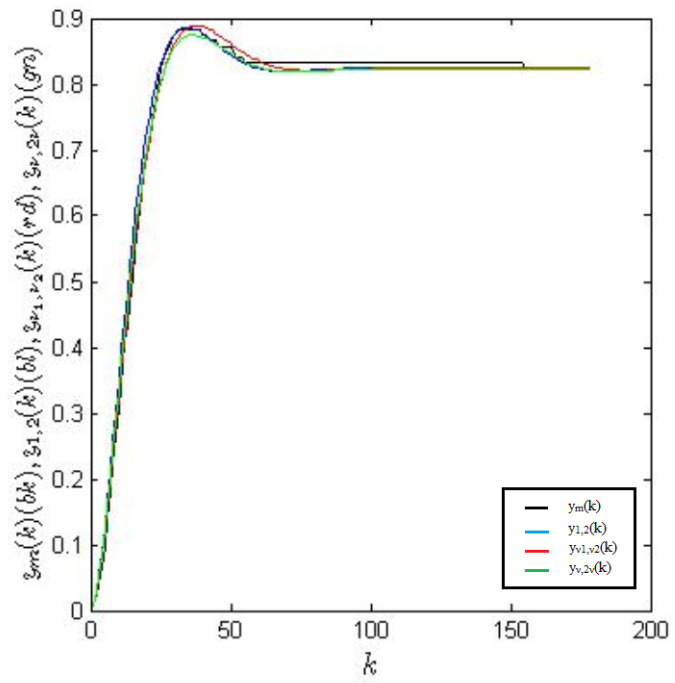
Plots of the responses: measured $y_{m}\left(k\right)$, simulated integer-order (IO) response $y_{1,2}\left(k\right)$ and fractional order (FO) models—non-commensurate $y_{\nu_{1},\nu_{2}}\left(k\right)$ and commensurate $y_{\nu ,2\nu }\left(k\right)$.

**Figure 8:**
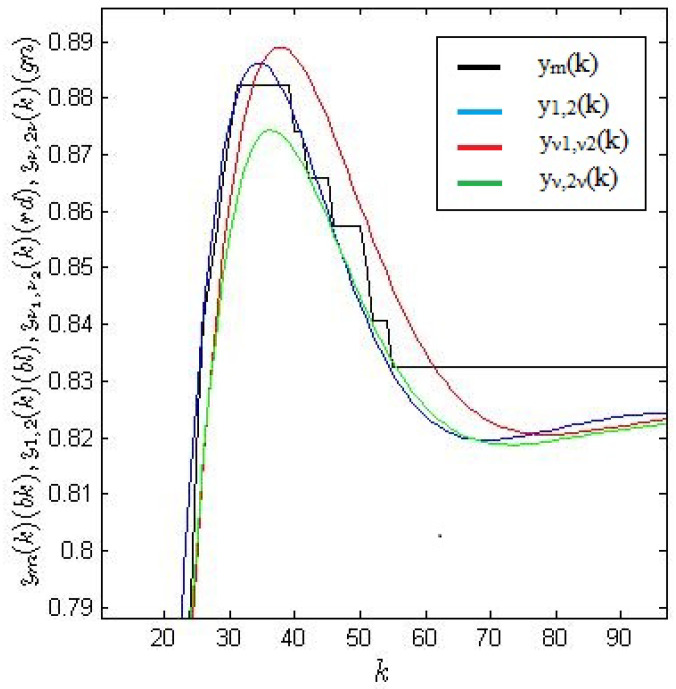
Enlarged fragment of Figure 7.

The plots of the measured output signal $y_{m}\left(kh\right)$ and the simulated responses $y_{2,1}$, $y_{\nu_{1},\nu_{2}}$ and $y_{\nu_{1},2\nu_{1}}$ are presented in Figure 6 and Figure 7, respectively. Figure 8 shows an enlarged fragment of Figure 7.

**Figure 9:**
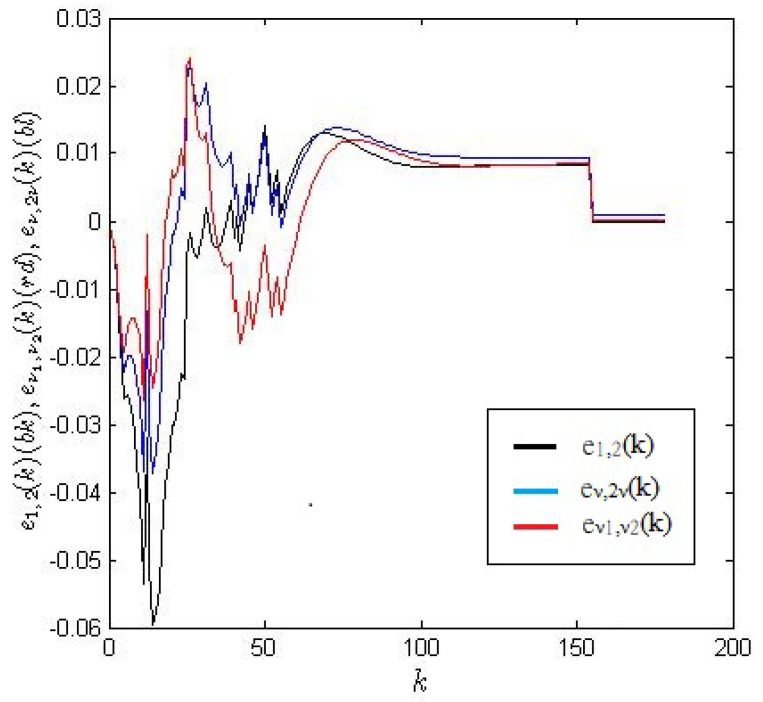
Plots of the errors $e_{1,2}\left(k\right)$ (black), $e_{\nu_{1},\nu_{2}}\left(k\right)$ (red) and $e_{\nu ,2\nu }$ (blue).

**Figure 10:**
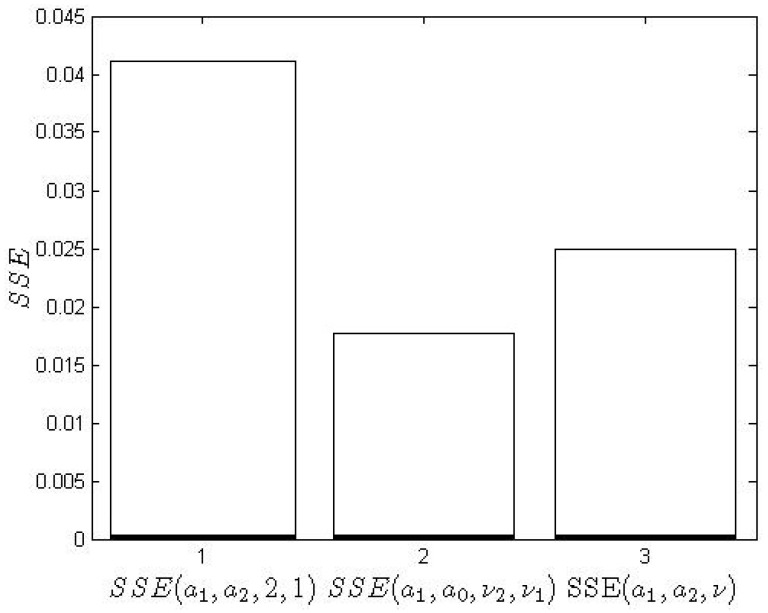
Minimum values of the sum of the squared error (SSE) criteria: $SSE(a_{0},a_{1})$, $SSE(a_{0},a_{1},\nu_{1},\nu_{2})$ and $SSE(a_{0},a_{1},\nu ,2\nu )$.

Figure 9 compares the errors resulting from the formulas (39)–(40). The values of the appropriate SSE are shown in Figure 10.

## 5. Conclusions

Numerical analysis shows that the application of FO models leads to an over 50% improvement in the SSE performance index compared to IO modeling. The commensurate model is inferior to the non-commensurate model, according to the relation

(45) $$\begin{array}{c} \min\left[SSE\left(a_{0},a_{1}\right)\right]\geq \min\left[SSE\left(a_{0},a_{1},\nu_{1}\right)\right]\\ \geq \min[SSE\left(a_{0},a_{1},\nu_{1},\nu_{2}\right)]. \end{array}$$

**Figure 11:**
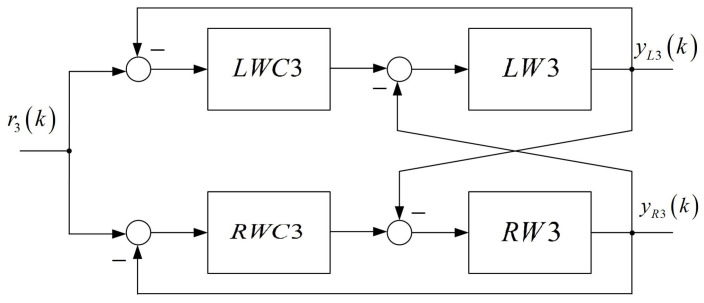
Block diagram of two cooperating wheels.

The proposed lowering of the total order is of particular importance in closed-loop systems with multiple inputs and multiple outputs with the same sub-plants. A block diagram of two cooperating wheels is shown in Figure 11. In the autonomous 6-wheel platform there are three such blocks.

In summary, the proposed fractional order approach leads to a good fit between the experimental data and the model formulation, proving that fractional calculus highlights aspects of dynamics that are to some extent overlooked from the standard integer order perspective.

## Figures and Tables

**Table 1:** Comparison of identification results.

Name	Model	$a_{1}$	$a_{0}$	$\nu_{1}$	$\nu_{2}$	SSE
IODE	(33)	0.1447	0.01447	1	2	0.0360
Non-commensurate FODE	(35)	0.145	0.01460	0.993	1.931	0.0175
Commensurate FODE	(37)	0.144	0.01456	0.983	1.966	0.0249
